# Alternatives to dental opioid prescribing after tooth extraction (ADOPT): protocol for a stepped wedge cluster randomized trial

**DOI:** 10.1186/s12903-024-04201-0

**Published:** 2024-04-04

**Authors:** Douglas R. Oyler, Philip M. Westgate, Sharon L. Walsh, Jennifer Dolly Prothro, Craig S. Miller, Monica F. Roberts, Patricia R. Freeman, Hannah K. Knudsen, Maggie Lang, Enif Dominguez-Fernandez, Marcia V. Rojas-Ramirez

**Affiliations:** 1 Department of Pharmacy Practice and Science, College of Pharmacy, University of Kentucky, 760 Press Avenue, Ste. 260, Lexington, KY 40536 USA; 2 Department of Biostatistics, College of Public Health, University of Kentucky, Lexington, KY USA; 3 Department of Behavioral Science and Center on Drug and Alcohol Research, College of Medicine, University of Kentucky, Lexington, KY USA; 4 Department of Oral Diagnosis, Medicine, and Radiology, College of Dentistry, University of Kentucky, Lexington, KY USA; 5 Substance Use Priority Research Area, University of Kentucky, Lexington, KY USA; 6 Department of Oral Health Science, College of Dentistry, University of Kentucky, Lexington, KY USA

**Keywords:** Analgesics, Opioid, Pain management, Dentists, Adolescent, Acetaminophen, Ibuprofen

## Abstract

**Background:**

Dentists and oral surgeons are leading prescribers of opioids to adolescents and young adults (AYA), who are at high risk for developing problematic opioid use after an initial exposure. Most opioids are prescribed after tooth extraction, but non-opioid analgesics provide similar analgesia and are recommended by multiple professional organizations.

**Methods:**

This multi-site stepped wedge cluster-randomized trial will assess whether a multicomponent behavioral intervention can influence opioid prescribing behavior among dentists and oral surgeons compared to usual practice. Across up to 12 clinical practices (clusters), up to 33 dentists/oral surgeons (provider participants) who perform tooth extractions for individuals 12–25 years old will be enrolled. After enrollment, all provider participants will receive the intervention at a time based on the sequence to which their cluster is randomized. The intervention consists of prescriber education via academic detailing plus provision of standardized patient post-extraction instructions and blister packs of acetaminophen and ibuprofen. Provider participants will dispense the blister packs and distribute the patient instructions at their discretion to AYA undergoing tooth extraction, with or without additional analgesics. The primary outcome is a binary, patient-level indicator of electronic post-extraction opioid prescription. Data for the primary outcome will be collected from the provider participant’s electronic health records quarterly throughout the study. Provider participants will complete a survey before and approximately 3 months after transitioning into the intervention condition to assess implementation outcomes. AYA patients undergoing tooth extraction will be offered a survey to assess pain control and satisfaction with pain management in the week after their extraction. Primary analyses will use generalized estimating equations to compare the binary patient-level indicator of being prescribed a post-extraction opioid in the intervention condition compared to usual practice. Secondary analyses will assess provider participants’ perceptions of feasibility and appropriateness of the intervention, and patient-reported pain control and satisfaction with pain management. Analyses will adjust for patient-level factors (e.g., sex, number of teeth extracted, etc.).

**Discussion:**

This real-world study will address an important need, providing information on the effectiveness of a multicomponent intervention at modifying dental prescribing behavior and reducing opioid prescriptions to AYA.

**ClinicalTrials.gov:**

NCT06275191.

**Supplementary Information:**

The online version contains supplementary material available at 10.1186/s12903-024-04201-0.

## Background

Reducing adolescent and young adult (AYA) substance use is a national priority [1]. Any prescription opioid use in or before high school is associated with future opioid misuse [2], and prescription opioid misuse by AYA is associated with future illicit drug use [3–5]. Strategies to reduce unnecessary opioid exposure among AYA are needed.

Dentists are a leading prescriber of opioids to AYA [6], and many of these prescriptions may be unnecessary. Between 65% and 70% of dental opioid prescriptions are issued after tooth extraction [7, 8], but individuals who use opioids to manage acute pain after a dental procedure do not report improved pain control or satisfaction with pain management [9]. In a comparison of over 58,000 patients with post-operative pain (almost exclusively after third molar extraction) across several studies, a combination of acetaminophen and ibuprofen was the most effective of the 46 analgesic regimens evaluated, including opioids [10]. Accordingly, non-opioid analgesics like acetaminophen and non-steroidal anti-inflammatory drugs (NSAIDs) are recommended as first-line analgesics for acute dental pain by the American Dental Association (ADA) [11], U.S. Centers for Disease Control and Prevention [12], and American Association of Oral and Maxillofacial Surgeons (AAOMS) [13]. 

Despite these recommendations, dentists and oral surgeons in the U.S. continue to prescribe opioids at rates much higher than in other countries [14, 15], and this may disproportionately affect AYA. Analysis of 44,387 individuals undergoing tooth extraction at the University of Kentucky (UK) College of Dentistry found that 41% of AYA patients received opioid prescriptions compared to 23% of patients from other age groups (*p* < 0.001) [16]. While opioid prescription likelihood is related to expected procedural characteristics (e.g., surgical extraction, number of teeth extracted, etc.), non-clinical variables such as day of the week and clinical practice site also increase the likelihood of opioid prescription [17, 18]. 

Together, the mismatch between existing practice (i.e., opioid prescribing despite recommendations to use more effective therapies) and the presence of non-clinical variables influencing practice suggest that comprehensive behavioral interventions to modify prescriber behavior are needed. The COM-B model for behavior change describes three primary drivers of behavior: capability (i.e., psychological and physical ability), opportunity (i.e., external factors), and motivation (i.e., conscious and unconscious processes) [19]. In the context of opioid prescribing for acute pain management after dental procedures, these may be considered as [1] knowledge regarding risks and benefits of analgesics; [2] pressure to prescribe opioids, which we have previously established is related to patient expectations; [20] and [3] removing immediate barriers to acute pain management [21]. In that context, this study will combine three elements to create a multicomponent intervention to modify prescriber behavior: [1] prescriber education using academic detailing [22, 23], [2] standardized patient post-extraction instructions for distribution, and [3] blister packaged acetaminophen and ibuprofen for distribution.

Previous efforts to change dental providers’ opioid prescribing behaviors have had mixed results. In 2016, the University of Minnesota School of Dentistry implemented an opioid prescribing protocol with concomitant provider education emphasizing first-line ibuprofen use unless contraindicated [24]. Compared to the 15 months before implementation of the protocol, the 15 months after protocol implementation reduced opioid prescriptions by 47.1% (2,792 prescriptions after vs. 5,279 prescriptions before). Teoh, et al. reported that combining a single group provider education session with an online dental prescribing reference reduced inappropriate antibiotic or opioid prescriptions from 130 (in the 6 weeks before the intervention) to 72 (in the 6 weeks after the intervention [25] supporting a general role for dentist/oral surgeon education to modify behavior. However, the recently published De-Implementing Opioids for Dental Extractions study found that a combination of clinical decision support and patient education did not change opioid prescribing across 49 providers in 22 clinics in Minnesota [odds ratio (OR) 1.27, 95% confidence interval (CI) 0.86–1.79, *p* = 0.18 compared to usual practice], largely due to low use of the clinical decision support tool by oral surgeons [26]. This suggests that interventions may need to be more comprehensive and implemented at the site, rather than individual, level (i.e., clinics instead of individual providers) to improve adherence and accountability.

One additive behavioral intervention that may change prescribing behaviors is providing prescribers with physical resources to issue instead of writing a new prescription. While this strategy has not been studied in dentists/oral surgeons performing tooth extractions, studies from other domains show promise. For example, the addition of generic drug vouchers (i.e., vouchers providing coverage for a 30-day supply of generic medication) to academic detailing increased prescribing of generic medications from 53.4 to 60.8% by 53 primary care physicians across 9 clinics [27]. Additionally, the use of drug samples (i.e., physical medication for distribution, a common strategy employed by pharmaceutical companies to increase prescribing of new medications) as described by Shrank and colleagues [28] has been associated with additive behavior change when compared to academic detailing alone. A claims analysis of 651 physicians who received academic detailing found that providing samples for generic atorvastatin was associated with a 2.6% increase in market share for the drug compared to other statin medications [29]. Similarly, a survey of 206 clinicians with (*N* = 148) and without (*N* = 58) industry-provided drug samples (i.e., samples for new brand-name medications) available in their practices found that sample availability influenced behavior: providers with samples available were significantly less likely to prescribe first-line generic therapies in standardized case vignettes [30]. Another study of over 2.3 million managed care organization beneficiaries found that providing generic first-line antibiotic drug samples to prescribers increased the prescribing rate of first-line antibiotics [31]. Finally, blister-packaged acetaminophen and ibuprofen has been provided to patients after tooth extraction previously, but the effect on provider behavior has not been measured. In a 2020 study, Derefinko, et al. randomized 76 adult patients undergoing dental surgery to an opioid misuse prevention program (*n* = 34) or treatment as usual (*n* = 32) [32]. The opioid misuse prevention program consisted of a 10-minute patient education session presented by an interventionalist plus a 7-day supply of acetaminophen and ibuprofen added to usual care. Although 95% of patients in both cohorts received an opioid prescription, patients who received the intervention reduced self-reported total opioid use in the week after surgery (38 vs. 48 total morphine milligram equivalents).

The primary objective of this study is to establish the effectiveness of the multicomponent intervention (academic detailing, patient education, and blister-packaged non-opioid analgesics) at reducing opioid prescriptions following tooth extraction among AYAs. The study will use a prospective, stepped wedge, cluster randomized trial (SW-CRT) design to assess the effectiveness of the intervention, which will be measured using encounter-level data from the electronic health record (EHR) to compare the odds of post-extraction opioid prescriptions to AYA under the intervention and control conditions. Secondary objectives are to examine implementation outcomes of the intervention (e.g., appropriateness and feasibility), and to assess AYA patient-reported pain management, satisfaction, and analgesic use after tooth extraction.

## Methods

This study is based on the standard protocol items: recommendations for interactive trials (SPIRIT).

### Study design

To optimize statistical power and allow each clinic to serve as an ethical control while ensuring each clinic is studied under the intervention condition, this study uses a flexible stepped wedge design. This novel design will allow for intervention start up in no more than two clusters at once.

**Fig. 1:**
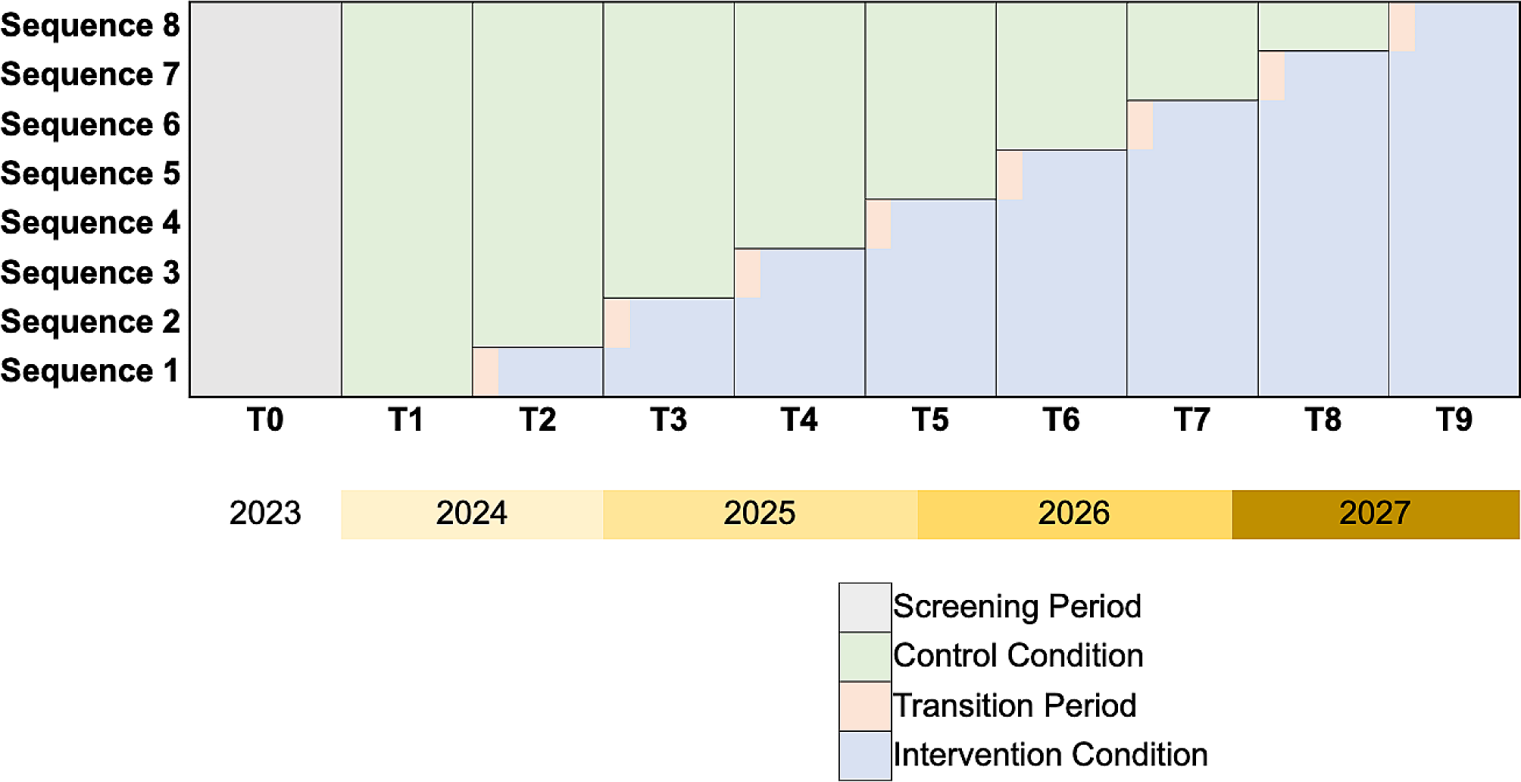
ADOPT study design. Stepped wedge schematic for the study assuming study initiation (T1) begins early 2024. Each ‘step’ from T1 to T9 will be 5 months in length. Shaded gray cells represent the baseline (T0) period during which clusters are screened for eligibility. Shaded green cells represent the control condition of usual care. Shaded peach cells represent the 1-month transition period, during which provider participants undergo the academic detailing session. Shaded blue cells represent the intervention condition, where provider participants distribute standardized post-extraction instructions and blister packs

Consistent with revised Consolidated Standards of Reporting Trials (CONSORT) guidelines for reporting SW-CRTs [33], this study is a repeated cross-sectional SW-CRT involving a sequential crossover of clusters from the control condition to the intervention condition, so that every cluster begins in the control condition and eventually receives the intervention condition. The period duration is 5 months with a 1-month transition period between the control and intervention conditions. The study uses 8 sequences, and each cluster is randomly assigned to a specific sequence. A schematic of the study design is presented in Fig. 1.

The cluster unit for this study is the participating dental or oral surgery practice. Each cluster contains a unique group of providers that can practice at one or more physical clinic locations, but may not practice in another cluster (i.e., a given cluster may consist of more than one physical clinic location if a practice has multiple offices). Up to 12 clusters may be included (accrual goal: *n* = 8), including up to 33 dentists (accrual goal: *n* = 24).

Patients (up to *n* = 50,000; minimum accrual goal: *n* = 5,040) will be exposed to the study condition of their dental clinic based on the date of their appointment. Opioid prescribing, measured at the encounter level from the electronic health record (EHR), will serve as the primary outcome. Additional implementation outcomes of appropriateness and feasibility will be measured at the provider level, and patient-reported pain-related outcomes will be measured using patient surveys.

Randomization to a specific sequence occurs at the cluster level to minimize potential contamination effects between providers at the same practice being randomized to receive the intervention at different times. The University of Kentucky (UK) institutional review board reviewed and approved this study.

### Study setting

Participating clinics include a mixture of academic and community settings across Kentucky and Southern Indiana, accounting for nearly 10,000 AYA tooth extractions annually. Each participating clinic uses EHRs and electronic controlled substance prescribing, and specific data queries have been developed for each EHR (WinOMS, axiUm, and Epic). The UK Biomedical Informatics Core at the Center for Clinical and Translational Science (CCTS) serves as the primary operational support to facilitate data sharing and storage in the UK Enterprise Data Warehouse, with additional logistical support provided by the UK Investigational Drug Service and Center on Drug and Alcohol Research. A full list of participating clinic sites can be found at clinicaltrials.gov.

### Eligibility criteria

Study-eligible clusters are dental or oral surgery practices that (a) performed permanent tooth extractions on at least 70 AYA in the baseline screening period of 5 months (from July 2022-November 2022) and (b) prescribed opioids in at least 30% of these AYA tooth extractions. Study-eligible providers are practicing dentists or oral surgeons at a study-eligible cluster. Study-eligible dental patients are aged 12–25 years that have a permanent tooth extraction performed by a study-eligible provider during the study period; the survey must be completed between post-extraction days 6 and 10. There are no additional exclusion criteria.

### Interventions

**Fig. 2:**
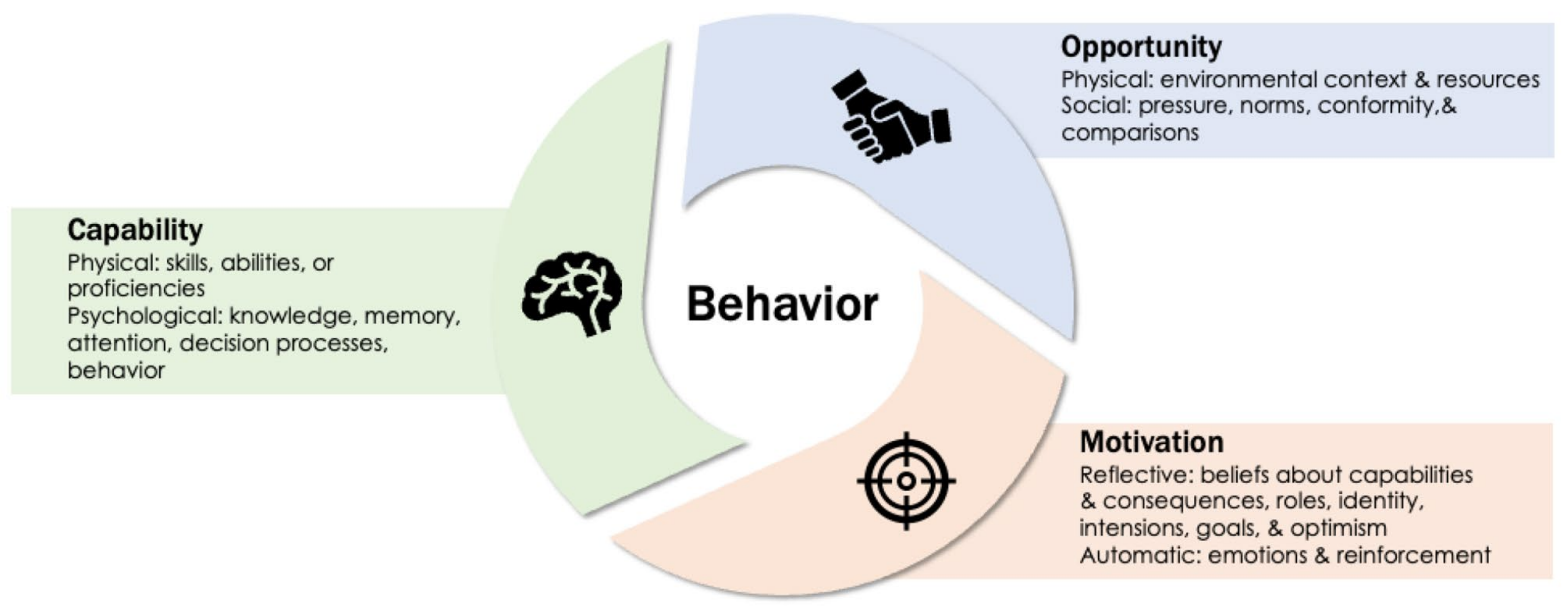
COM-B theoretical framework. Capability, Opportunity, and Motivation for Behavior Change (COM-B) model, adapted from Michie, et al. [19] As applied to the study intervention, academic detailing aims to increase physical capability, psychological capability, and automatic motivation; revised patient instructions aim to reduce social pressures and reflective motivation; and blister-packaged analgesics aim to provide a physical resource to increase opportunity and reflective motivation

The study intervention follows the COM-B model for behavior change as outlined in Fig. 2. The intervention is composed of three elements: academic detailing, provision of standardized post-extraction patient instructions, and provision of blister-packaged acetaminophen and ibuprofen for distribution.

#### Academic detailing

During the transition period, providers will receive at least one 45-to-60 min in-person or video-assisted academic detailing session with an academic detailing pharmacist trained by the National Resource Center for Academic Detailing. Academic detailing will build on the safer opioid prescribing for acute dental clinics strategy from the Opioid-overdose Reduction Continuum of Continuum of Care Approach [34], applying principles and methodology from the Centers for Disease Control and Prevention Academic Detailing to Enhance Overdose Prevention implementation guide [35]. Specifically, the format of the academic detailing visit will follow the Introduction; Needs Assessment; Key Messages, Features, and Benefits; Barriers and Objections; and Summary and Close steps outlined in the CDC implementation guide. Academic detailing for the ADOPT study will focus on 5 key messages:

1. Use acetaminophen and ibuprofen first line for pain management.
2. Carefully consider risk factors for overdose when prescribing opioids.
3. Warn patients and parents about misuse when prescribing opioids to AYA.
4. Establish realistic expectations and help every patient understand the pain management plan.
5. Keep your community safe with medication disposal education.

Academic detailing materials were developed by the study team based on materials used in the HEALing (Helping to End Addiction Long Term^SM^) Communities Study-Kentucky [34] and were peer-reviewed in collaboration with AAOMS.

#### Standardized post-extraction patient instructions

Distribution of post-extraction instructions after tooth extraction is usual care. In the intervention condition, provider participants will receive standardized paper post-extraction patient instructions for distribution. These materials were developed by the study team based on existing patient education materials at participating clinics and the ADA’s Tooth Extraction: Post-Operative Instructions brochure, and materials were peer-reviewed in collaboration with AAOMS. Specific messages in these materials regarding pain management mirror relevant components of academic detailing sessions, specifically focusing on taking medications as directed, using acetaminophen and ibuprofen first, and how to dispose of unused opioids (if prescribed) based on available community resources (i.e., local take-back locations). Additionally, patient instructional materials include information consistent with labeling of acetaminophen and ibuprofen.

In the intervention condition, provider participants (or other clinical staff specific to a given participating clinic’s standard workflow) can distribute the standardized patient post-extraction instructions instead of the usual post-extraction instruction.

#### Standardized, pre-packaged Acetaminophen and ibuprofen blister packs

In the intervention condition, provider participants will receive standardized, pre-packaged blister packs containing a 7-day supply of acetaminophen 500 mg and ibuprofen 400 mg for oral use every 6 h (i.e., 28 tablets of each medication in a single 7-day pack). In the intervention condition, provider participants (or other clinical staff specific to a given participating clinic’s standard workflow) can distribute the standardized, pre-packaged blister packs consistent with clinical judgment in accordance with appropriate state medication dispensing regulations.

Acetaminophen is packaged as 500 mg per dose to be administered four times daily (total 2,000 mg per day). This dose is as effective as higher doses [10]. Use of a 500 mg dose administered 4 times per day results in a total daily dose below the daily maximum recommended by the U.S. Food and Drug Administration (4,000 mg) and ADA (3,000 mg). Additionally, this dose can be used across the entire patient population targeted by this study, as this is acceptable pediatric dosing for any individual weighing at least 27 kg, which is the 1st percentile for weight for 12-year-old boys and 0th percentile for weight for all other age/sex groups. As opioid prescriptions can still be provided, 2,000 mg total daily dose of acetaminophen allows for the most common opioid prescription issued (hydrocodone with acetaminophen 5/325 mg every 6 h) [7] to be co-administered while still staying under the maximum recommended total daily dose of acetaminophen.

Ibuprofen will be packaged as 400 mg per dose to be administered four times daily (total 1,600 mg per day). This dose is as effective as higher doses and more effective than lower doses [10, 36]. Multiple studies indicate a favorable safety profile of ibuprofen and no significant increase in risk of gastrointestinal, cardiovascular, renal, or hepatic adverse reactions at this dose [37–40], which is acceptable dosing for the entire patient population targeted by this study.

### Outcomes

Primary and secondary outcomes are listed in Table 1. The primary outcome measure, AYA receipt of an opioid prescription after tooth extraction, will be compared between intervention and control conditions using EHR extracts from each participating clinic. Secondary outcomes are assessed using electronic surveys and include provider participant assessment of intervention implementation feasibility and appropriateness (2a, 2b), as well as patient-reported pain intensity (2c), interference (2d), and satisfaction (2e).

**Table 1:** Objectives, endpoints, and data sources

Objectives	Endpoint	Data Source
**Primary**		
To compare the odds of post-extraction opioid prescriptions to AYA under the intervention condition relative to the control condition	Study condition comparison of the patient-level odds of post-extraction opioid prescription	EHR
**Secondary**		
To compare provider-perceived intervention appropriateness and feasibility before and after intervention implementation	Differential pre- to post-intervention change by provider in the rating of intervention feasibility and appropriateness	Provider survey before and after transition period
To compare provider-perceived appropriateness and feasibility of decreasing opioid prescribing to AYA before and after intervention implementation	Differential pre- to post-intervention change by provider in the rating of opioid prescribing reduction feasibility and appropriateness	Provider survey before and after transition period
To compare AYA self-reported pain level within 10 days after tooth extraction based on self-reported opioid use.	Comparison of worst, least, and average pain over the 10 days after extraction based on self-reported opioid use	Patient survey within 6–10 days after tooth extraction
To compare AYA self-reported pain interference within 10 days after tooth extraction based on self-reported opioid use.	Comparison of average pain interference T-score (NIH PROMIS Pediatric Short Form v1.0 – Pain Interference 8a, or NIH PROMIS Adult Short Form v1.0 – Pain Interference 6b) based on self-reported opioid use.	Patient survey within 6–10 days after tooth extraction
To compare AYA self-reported satisfaction with pain management within 10 days after tooth extraction based on self-reported opioid use	Comparison of average overall pain satisfaction based on self-reported opioid use.	Patient survey within 6–10 days after tooth extraction

AYA, adolescent and young adult; EHR, electronic health record

### Statistical analysis

Analyses comparing intervention (academic detailing and provision of patient post-extraction instructions and blister packs for distribution) to usual practice (control condition) will be based on the intent-to-treat approach. Specifically, any given patient’s observations in analyses will correspond to the trial condition to which their cluster was randomized during the given period. Analyses will be conducted in SAS 9.4 or higher and/or R, and all statistical tests will be two-sided with a significance level of 0.05.

#### Primary outcome

The primary outcome is a patient-level binary indicator for being prescribed an opioid. Generalized estimating equations (GEE) will be used to fit a population-averaged, or marginal, model to compare the odds of opioid prescribing between intervention and control conditions, accounting for the statistical correlation among outcomes from the same cluster [41–43]. The primary statistical test will be the test of trial condition to determine if the intervention decreases the odds of opioid prescribing. Furthermore, in accordance with statistical modeling expectations for the analysis of data arising from stepped wedge cluster randomized trials, our model will adjust for period such that time will not confound the estimated impact of the intervention [41, 44, 45]. PROC GLIMMIX in SAS will be used to fit the marginal logistic regression model [43, 46]. To ensure valid inference, small-sample adjustments to standard error estimates, and the use of degrees of freedom equaling the number of clusters minus two, will be utilized [42]. 

#### Secondary outcomes

Analyses of secondary objectives 1 and 2 are meant to be exploratory in nature. Therefore, we will assess pre-transition survey and post-transition survey scores based on descriptive statistics (e.g., sample median, sample size, etc.). However, we will formally test for differences based on appropriate paired tests. Specifically, because all survey items will be ranked using a numerical 5-point Likert scale, we anticipate Wilcoxon signed-rank tests on the pairs will be utilized. However, paired t-tests or mixed models will first be considered if assumptions are adequately met to improve power. To account for statistical correlation among outcomes from provider participants at the same cluster with paired tests, provider participant outcomes will first be averaged over each cluster for any given time point. Secondary objectives 1 and 2 are each addressed via two sub-objectives, each utilizing 4 Likert-type questions, and hence corresponding analyses as described above. To account for multiple testing within sub-objectives, *p*-values will be adjusted based on the false discovery rate [47]. 

Secondary objectives 3, 4, and 5 compare patient participants who were and who were not prescribed opioids, based on self-report, as opposed to comparing intervention to control conditions. Therefore, the independent variable of interest in statistical modeling for this aim is a subject-level indicator for having been prescribed opioids. However, models will still adjust for the trial arm condition based on randomization, and hence account for the intention-to-treat principle, as well as period and adjusting for patient-level factors. All available data will be utilized. Scores for these objectives will be treated as continuous patient participant-level outcomes and analyzed using linear mixed effects models. PROC GLIMMIX in SAS will be used to fit the models.

### Sample size

This study is powered based on the primary outcome. Power calculations are based on 8 clusters being randomized to 8 different sequences corresponding to the stepped-wedge design. Based on historic pilot data, at least 70 patients will be observed per cluster per period. At least 41% of patients in the control condition are expected to receive an opioid prescription. Based on the effectiveness of individual intervention components, the intervention should decrease the marginal percentage by at least 18% (i.e., from 41 to 23%).

Power calculations consider the intra-cluster correlation coefficient (ICC) and inter-period correlation. The ICC, or within-period correlation among subject-level opioid prescription outcomes from within the same cluster during the same period, is conservatively estimated at 0.10 [48, 49]. Assuming the ICC and inter-period correlations are equivalent, this study has over 99% power to detect the intervention effect [41, 44]. Furthermore, conservatively assuming no inter-period correlation, this study has at least 86% power.

### Participant recruitment and informed consent

Providers, oftentimes the owners of eligible clusters, have been engaged in cluster recruitment. Full informed consent from providers will be obtained prior to enrollment. Consistent with the stepped wedge design, all clusters (and thus all providers) will begin the study in the control condition and will transition to the intervention condition based on the sequence to which they are randomized.

For the survey-based secondary study objectives, QR-coded signage will be placed in high-traffic areas in participating clinics inviting AYA patients to complete the survey. QR codes may also be distributed with patient materials, based on the workflow at each clinic. As dental patients are only eligible between post-extraction days 6 and 10, patients who attempt to access the survey early (e.g., at or before the extraction appointment) will be invited to receive an automated text or email reminder to complete the survey on post-extraction day 7. Stratified sampling will not be used because the secondary survey objectives are not directly related to the intervention. A survey cover letter will be used in lieu of full informed consent for survey participation.

### Randomization

The study statistician will randomize between 8 and 12 clusters to the 8 sequences via a random permutation. If more than 8 clusters are enrolled, additional clusters will be randomized to sequences 4, 5, 3, and 6 (Fig. 1). The study statistician will notify investigators of the next cluster to transition at the end of each period.

### Data collection

EHR data will be utilized for the primary objective. EHR extracts will be uploaded by participating clinics quarterly using a secure file transfer process managed by the UK Institute for Biomedical Informatics. EHR extracts will contain analgesic prescriptions and associated characteristics (e.g., medication name, strength, directions, and quantity), as well as patient-level variables including demographics, number and site of teeth extracted, and extraction method.

Secondary objectives will be assessed by provider and participant surveys conducted using Research Electronic Data Capture (REDCap) tools hosted at UK [50, 51]. Pre- and post-transition provider participant surveys will be administered at the final visit in the control condition, approximately 1 month prior to the transition period, and at the third visit in the intervention condition approximately 3 months after the transition period. The surveys each contain between 17 and 21 total questions (based on branching logic), are completed electronically, and take less than 5 min to complete. The survey instrument was developed based on the validated instrument from Weiner, et al. to assess acceptability, appropriateness, and feasibility of implementation measures using a 5-point Likert scale from completely agree to completely disagree. Based on feedback obtained during a pilot of the survey with 5 dentists, acceptability measures were removed from the final survey instrument.

Patient survey data will be collected electronically using a REDCap survey completed between post-extraction days 6 and 10. The patient survey instrument contains between 19 and 27 questions (based on branching logic) and takes approximately 5 min to complete. The survey was modeled after a previously published survey of 329 patients (average age 41–52 years) undergoing tooth extraction. The instrument for this study was modified to reduce Flesh-Kincaid reading level to 6.3 and added pain interference, pain intensity, and medication use questions. Pain interference is assessed using NIH PROMIS Pediatric Short Form v1.0 – Pain Interference 8a or NIH PROMIS Adult Short Form v1.0 – Pain Interference 6b, based on a respondent age cutoff of 18 years. Pain intensity is measured using the Brief Pain Inventory. The instrument used for this study was piloted with 8 patients aged 12–25 years undergoing tooth extraction at UK.

### Missing data

For the primary outcome, patients are not followed over time, so no missing outcome data are expected, and analyses will use all available data. Similarly, regarding secondary endpoints from patients and provider participants, all available data will be utilized. Although unlikely, if a cluster or provider drops out of the study, all available data to the point of withdrawal will be used.

### Data management

Data collection and accurate documentation are the responsibility of the research personnel under the supervision of the principal investigators. All source documents and reports will be reviewed by the study team and data entry staff to ensure accuracy and completeness. All EHR data shared via the secure file transfer will be de-identified and uploaded to a secure server folder in the UK Enterprise Data Warehouse by an honest broker affiliated with UK CCTS. Access to the secure folder will be limited to members of the biostatistical study team, with analysis using the CCTS Virtual Machine. Survey data will be collected in REDCap, stored on secure REDCap servers in the UK CCTS, only accessible to members of the study team. Data analysis will be conducted in accordance with the statistical analysis plan.

### Quality control and fidelity monitoring

Study personnel are trained to conduct study visits using Meeting Guides, which provide scripted verbiage and checklists to ensure consistent protocol implementation and barrier assessments across clinics and participants. Quality assurance reviews are conducted weekly, with study-wide summary reports compiled on a semiannual and annual basis. Corrective action plans are devised from findings and progress is reviewed monthly.

Quality and fidelity of the academic detailing session is monitored using electronic case report forms in the REDCap database. Fidelity achievement for academic detailing visits is defined as a visit lasting at least 30 min with delivery of at least 3 key messages.

Distribution of patient post-extraction instruction materials and blister packs is at the discretion of the provider participant. Clinics will record materials provided to patients using a distribution monitoring log. The log is reviewed during routine visits with study staff, which occur at least quarterly throughout the study, and reconciled with EHR data extracts to proactively address barriers to distribution. Individuals completing the patient survey during the intervention condition are asked about receipt of blister packs to allow for additional fidelity monitoring.

### Clinic workflow considerations

All reasonable efforts to remove participation barriers will be attempted throughout the study. As the unit of randomization is the clinic (vs. the individual provider), only a single workflow for the clinic is required. Additionally, study staff will conduct on-site visits with participating clinics at least quarterly to assess barriers to participation. Data collection relies primarily on EHR extracts, which requires no additional real-time study documentation and has been customized to and piloted with each potential clinic prior to study enrollment.

### Safety assessments

This study does not include objectives or endpoints concerning safety. However, monitoring activities, including active assessment of potential adverse events in the patient survey, will be conducted to assess possible harm.

## Discussion

Opioids continue to be frequently prescribed after tooth extractions in the United States. Given many of these extractions are performed in AYA, a population at particularly high risk of developing problematic opioid use, strategies are needed to reduce unnecessary opioid exposure. It is well-established that nonopioid analgesics such as acetaminophen and ibuprofen provide similar analgesia, so strategies to translate research to practice are necessary. This trial examines the effectiveness of a targeted strategy built around an established model of behavior change (COM-B) to facilitate guideline-concordant opioid prescribing. Additionally, this study uses established implementation frameworks to assess appropriateness and feasibility of both the intervention and changing prescribing and expands on existing patient satisfaction data to an AYA cohort. Thus, the findings from this study could identify effective, scalable means to change practice, improve patient care, and reduce unnecessary risk.

### Trial status

The ADOPT study is a current, ongoing trial that is not yet recruiting study participants. Recruitment initiation is planned for March 2024, with the study conducted from March 2024 through November 2027. Results will be presented in 2028.

### Electronic supplementary material

Below is the link to the electronic supplementary material.

Supplementary Material 1

## Data Availability

Results generated from this study will be available at clinicaltrials.gov at the completion of the study. Underlying primary data will be deposited for sharing in the National Addiction & HIV Data Archive Program. Other study materials will be deposited for sharing in the Open Science Foundation repository.

## Abbreviations

AAOMS: American Association of Oral and Maxillofacial Surgeons
ADA: American Dental Association
AYA: Adolescent and young adult
CCTS: Center for Clinical and Translational Science
COM-B: Capability, Opportunity, and Motivation?Behavior (Model)
CONSORT: Consolidated Standards of Reporting Trials
HER: Electronic health record
GEE: Generalized estimating equations
HEALing: Helping End Addiction Long-Term
ICC: Intra-cluster correlation coefficient
NSAID: Nonsteroidal anti-inflammatory drug
REDCap: Research Electronic Data Capture
SW-CRT: Stepped wedge cluster randomized trial
UK: University of Kentucky
