# Supplementary material for: Alternatives to dental opioid prescribing after tooth extraction (ADOPT): protocol for a stepped wedge cluster randomized trial
Source: BMC Oral Health. 2024 Apr 4;24:414. doi: 10.1186/s12903-024-04201-0 (PMC10996080; doi:10.1186/s12903-024-04201-0)
Supplement: Supplementary file 1 — Supplementary Material 1 [file 12903_2024_4201_MOESM1_ESM.docx]

Appendix. World Health Organization Trial Registration Data Set Elements

| **Data Category** | **Information** |
| --- | --- |
| Primary registry and trial identifying number | ClinicalTrials.gov  NCT06275191 |
| Date of registration in primary registry | 22 February, 2024 |
| Secondary identifying numbers | None |
| Source(s) of monetary or material support | National Institute of Dental and Craniofacial Research |
| Primary sponsor | National Institute of Dental and Craniofacial Research |
| Secondary sponsor | None |
| Contact for public queries | JDP [adoptstudy@uky.edu] |
| Contact for scientific queries | DO [doug.oyler@uky.edu] |
| Public title | Alternatives to Dental Opioid Prescribing After Tooth Extraction (ADOPT) |
| Scientific title | Alternatives to Dental Opioid Prescribing After Tooth Extraction (ADOPT) |
| Countries of recruitment | United States of America |
| Health condition(s) or problem(s) studied | Analgesics, Opioid; Acute Pain; Tooth Extraction; Adolescent; Acetaminophen; Ibuprofen |
| Intervention(s) | Behavioral: Multicomponent intervention  The multicomponent intervention consists of (1) a single 45-60-minute academic detailing session with provider participants, plus (2) provision of patient post-extraction instruction materials and (3) provision of blister-packaged acetaminophen and ibuprofen for distribution to adolescent/young adult patients after tooth extraction in the course of clinical practice |
| Key inclusion and exclusion criteria | Ages eligible for study: all (provider); 12-25 (patient)  Sexes eligible for study: all  Accepts healthy volunteers: no |
|  | Inclusion Criteria (Provider):   - Actively U.S. licensed dentist or oral surgeon practicing at a participating cluster. Participating clinics were finalized prior to the beginning of the study based on the following criteria: (1) perform tooth extractions on at least 70 AYA patients during a 5-month screening period from July 1, 2022 through November 30, 2022 and (2) electronically prescribe opioids to at least 30% of AYA patients who underwent extraction during the screening period. Additionally, each participating cluster signed a data use agreement (DUA) for sharing electronic health record data, can provide the necessary electronic health record data, and agreed to comply with study procedures and be available for the duration of the study. - Provide a signed and dated informed consent form. - Willing to comply with all study procedures and be available for the duration of the study.   Inclusion Criteria (Patient):   - Undergoes any tooth extraction at a participating cluster during the study (intervention condition, transition period, or control condition) between 6-10 days earlier. - Age 12-25 at the time of tooth extraction. - Reads and acknowledges survey cover letter in lieu of a full consent/assent process. - Can access the electronic survey using an internet-capable device. |
|  | Exclusion Criteria:   - There are no other exclusion criteria for clusters or provider/patient participants that meet all inclusion criteria. |
| Study type | Interventional |
|  | Allocation: Randomized. Assignment masking: double (participant, investigator). |
|  | Primary purpose: other |
|  | Phase: N/A |
| Date of first enrollment | April 2024 [anticipated] |
| Target sample size | 38159 [anticipated] |
| Recruitment status | Not yet recruiting |
| Primary outcome(s) | Odds of post-extraction opioid prescription to adolescent/young adults after tooth extraction  The primary outcome is a patient-level binary indicator for being prescribed an opioid. The value for this outcome variable is equal to 1 if the patient is prescribed an opioid, whereas this variable takes on a value of 0 if this patient is not prescribed an opioid. Opioid prescription will be defined as an electronic order (from the electronic health record) for an opioid analgesic (e.g., hydrocodone, oxycodone, tramadol, morphine, fentanyl, etc.) on the same calendar date as the tooth extraction appointment.  [Time Frame: Same calendar date as dental extraction. Electronic health records will be extracted quarterly for the duration of the 4-year study period.] |
| Key secondary outcomes | 1. (Change in) intervention feasibility   Survey response. 5-point Likert-type questions (from 1-completely disagree to 5-completely agree) regarding feasibility of distributing blister packs and patient post-extraction materials. Selection of the top 2 choices (completely agree or agree) from the scale is considered as agreement with the statement.  [Time Frame: Pre-surveys will be conducted during the calendar month preceding the transition to the intervention condition (i.e., within 60 days). Post-surveys will be conducted during the 3rd month of the intervention condition (i.e., within 120 days).]   1. (Change in) intervention appropriateness   Survey response. 5-point Likert-type questions (from 1-completely disagree to 5-completely agree) regarding appropriateness of distributing blister packs and patient post-extraction materials. Selection of the top 2 choices (completely agree or agree) from the scale is considered as agreement with the statement.  [Time Frame: Pre-surveys will be conducted during the calendar month preceding the transition to the intervention condition (i.e., within 60 days). Post-surveys will be conducted during the 3rd month of the intervention condition (i.e., within 120 days).]   1. (Change in) opioid prescribing feasibility   Survey response. 5-point Likert-type questions (from 1-completely disagree to 5-completely agree) regarding feasibility of reducing opioid prescription. Selection of the top 2 choices (completely agree or agree) from the scale is considered as agreement with the statement.  [Time Frame: Pre-surveys will be conducted during the calendar month preceding the transition to the intervention condition (i.e., within 60 days). Post-surveys will be conducted during the 3rd month of the intervention condition (i.e., within 120 days).]   1. (Change in) opioid prescribing appropriateness   Survey response. 5-point Likert-type questions (from 1-completely disagree to 5-completely agree) regarding appropriateness of reducing opioid prescription. Selection of the top 2 choices (completely agree or agree) from the scale is considered as agreement with the statement.  [Time Frame: Pre-surveys will be conducted during the calendar month preceding the transition to the intervention condition (i.e., within 60 days). Post-surveys will be conducted during the 3rd month of the intervention condition (i.e., within 120 days).]   1. Self-reported pain   Patient survey, 3 items from Brief Pain Inventory (worst, least, average pain) based on self-reported opioid use. Each item uses an 11-point Visual Rating Scale from 0-no pain to 10-worst possible pain.  [Time Frame: within 10 days of tooth extraction]   1. Self-reported pain interference   Patient survey, NIH PROMIS Pediatric Short Form v1.0-Pain Interference 8a (age < 18) or NIH PROMIS Adult Short Form v1.0-Pain Interference 6b (18+) based on self-reported opioid use. NIH PROMIS Pediatric Short Form t-scores range from 34.0 to 78.0, with higher scores indicating greater interference. NIH PROMIS Adult Short Form t scores range from 41.0 to 78.3, with higher scores indicating greater interference.  [Time Frame: within 10 days of tooth extraction]   1. Self-reported pain satisfaction   Patient survey, 5-point Likert-type question (from 1-very unhappy to 5-very happy) regarding overall satisfaction with pain management based on self-reported opioid use. Selection of the top 2 choices (very happy or happy) from the scale is considered as satisfaction with overall pain management.  [Time Frame: within 10 days of tooth extraction] |

Protocol Version Number: 1.0 (19 February 2024)
